# The Dynamics of Calcium Signaling in Beta Cells—A Discussion on the Comparison of Experimental and Modelling Data

**DOI:** 10.3390/ijms24043206

**Published:** 2023-02-06

**Authors:** Michael Müller, Jonas Walkling, Nele Seemann, Ingo Rustenbeck

**Affiliations:** 1 Institute of Dynamics and Vibrations, Technische Universität Braunschweig, D-38106 Braunschweig, Germany; 2 Institute of Pharmacology, Toxicology and Clinical Pharmacy, Technische Universität Braunschweig, D-38106 Braunschweig, Germany

**Keywords:** calcium signaling, membrane potential, insulin secretion, integrated oscillator model, measurement feedback, parameter sensitivity, simulation

## Abstract

The stimulus–secretion coupling of the pancreatic beta cell is particularly complex, as it integrates the availability of glucose and other nutrients with the neuronal and hormonal input to generate rates of insulin secretion that are appropriate for the entire organism. It is beyond dispute however, that the cytosolic Ca^2+^ concentration plays a particularly prominent role in this process, as it not only triggers the fusion of insulin granules with the plasma membrane, but also regulates the metabolism of nutrient secretagogues and affects the function of ion channels and transporters. In order to obtain a better understanding of the interdependence of these processes and, ultimately, of the entire beta cell as a working system, models have been developed based on a set of nonlinear ordinary differential equations, and were tested and parametrized on a limited set of experiments. In the present investigation, we have used a recently published version of the beta cell model to test its ability to describe further measurements from our own experimentation and from the literature. The sensitivity of the parameters is quantified and discussed; furthermore, the possible influence of the measuring technique is taken into account. The model proved to be powerful in correctly describing the depolarization pattern in response to glucose and the reaction of the cytosolic Ca^2+^ concentration to stepwise increases of the extracellular K^+^ concentration. Additionally, the membrane potential during a KATP channel block combined with a high extracellular K^+^ concentration could be reproduced. In some cases, however, a slight change of a single parameter led to an abrupt change in the cellular response, such as the generation of a Ca^2+^ oscillation with high amplitude and high frequency. This raises the question as to whether the beta cell may be a partially unstable system or whether further developments in modeling are needed to achieve a generally valid description of the stimulus–secretion coupling of the beta cell.

## 1. Introduction

### 1.1. Physiological Background 

The pancreatic beta cell transduces the increased availability of glucose and a few other carbohydrates, amino acids and keto acids into stimulated rates of secretion. While a number of neuronal and hormonal signals are able to modify the secretory response, it is the metabolic breakdown of these nutrients which produces the indispensable signals for stimulated secretion [1,2]. The KATP channel at the plasma membrane was identified as the link between the energy metabolism and the electrical activity of the beta cell, since the increased ATP/ADP ratio in the cytosol leads to a decreased potassium efflux, thus enabling the depolarization of the plasma membrane [3,4]. The view that the depolarization-induced Ca^2+^ influx via voltage-dependent Ca^2+^ channels is the final common pathway of stimulus–secretion coupling, and that the pattern of the cytosolic Ca^2+^ concentration determines the pattern of insulin secretion [5], was qualified by the identification of the amplifying pathway, which affects the secretion by signals circumventing plasma membrane depolarization [6,7]. 

Nevertheless, it is beyond dispute that the cytosolic Ca^2+^ concentration is of central importance not only for the onset of stimulated insulin secretion, but also for its entire duration. It regulates not only the fusion of insulin granules with the plasma membrane, but also the granule itinerary [8]. Additionally, the cytosolic Ca^2+^ concentration mediates multiple feedback loops by influencing the mitochondria, the endoplasmic reticulum and Ca^2+^-dependent K^+^ channels at the plasma membrane [9,10,11]. In view of the resultant complexity of the interrelations, modelling is necessary to achieve a quantitative description of the dynamics of the Ca^2+^ concentration in the cytosol and in subcellular compartments, together with changes in the membrane potential and in the glucose metabolism. This paper aims to compare published models to describe these dynamics, with actual measurements from the literature as well as from our own work. Because they are essential for the discussion, the established procedures for measuring and modelling these processes are subsequently presented in detail.

### 1.2. Methods for the Determination of the Plasma Membrane Potential 

**Figure A1:**
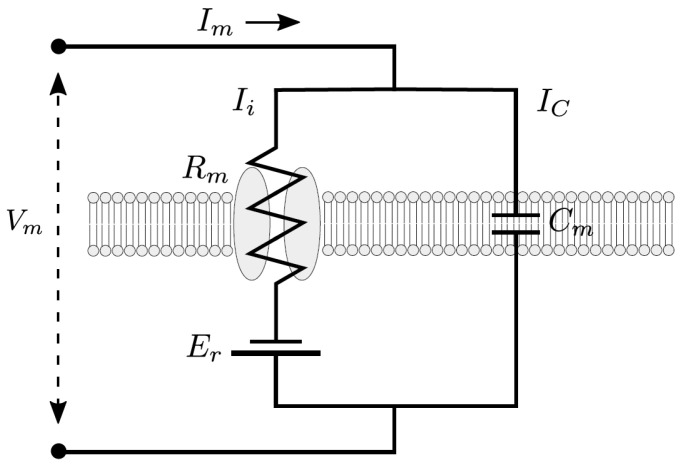
Simplified circuit for the electrical system on a cell membrane during the patch-clamp process.

While the measurement of the plasma membrane potential has also been performed with fluorescent indicators such as oxonol or carbocyanine dyes [12], more quantitative observations of electrical activity are obtained with electrophysiological methods (see Figure A1 in Appendix A). In the original version, a sharp microelectrode had to be inserted into the cell under study, and the voltage was measured with reference to another electrode in the surrounding solution. This voltage is then fed into a comparator and compared with a previously set target voltage (i.e., the “command potential”). The current required to maintain the desired voltage is injected via a second inserted electrode, and the course of the voltage between the cell and the surrounding medium is calculated from this measurement. In contrast to the voltage clamp, a current can also be preset to flow to the cell. This is achieved by dropping a predetermined potential across a known resistance. Since no voltage is specified, a current clamp measurement results [13].

The microelectrode technique reports the entirety of events at the plasma membrane [14,15], which is not a disadvantage for the measurement of the membrane potential, but precludes the analysis of ion currents at the single-channel level. This disadvantage was overcome by the development of the patch clamp technique [16]. In this method, the cell membrane is not perforated, but a smooth pipette is placed on the cell and a small separated area (or “patch”) is created by slight suction applied to the pipette. The close contact (or “gigaseal”) isolates the membrane patch from the rest of the cell and enables single-channel measurements [16]. The electrode for the measurement is located in the pipette and serves both purposes of voltage measurement with reference to ground and current injection. 

Further developments have widened the application of this technique beyond single-channel measurements. If stronger suction is applied to the attached cell, the membrane patch is ruptured and a direct access to the cell interior is generated. This configuration is referred to as “whole cell recording” [17], which corresponds to the measurement with an inserted sharp electrode but has a much larger connection between the pipette solution and the cytosol. To preclude the dialysis of the cytosol, the “perforated patch” configuration was devised, wherein the electrical contact between the pipette solution and the cell interior is created by pore-forming antibiotics such as nystatin [18]. 

### 1.3. Methods to Study the Cytosolic Calcium Concentration 

The early measurements of the intracellular Ca^2+^ concentration were performed with electrodes in glass pipettes that contained a Ca^2+^ ionophore solution in the tip [19]. This required the impaling of the cells under study and was a manually demanding technique. The decisive methodological advance was achieved by the synthesis of fluorescent indicators which change the fluorescence emission, depending on the coordinative binding of Ca^2+^. In order to load the cells with these indicators, the cells must be membrane permeant; this was achieved by masking the Ca^2+^ binding groups with acetoxymethyl esters. The ester bond can be cleaved by esterases in the cytosol, and the now polar indicator compound is trapped within the cell [20]. While the accumulation of the indicator is needed for a good signal-to-noise ratio, an unavoidable drawback is that the binding of Ca^2+^ can decrease its cytosolic concentration and thus affect the signal to be reported when the concentration of the indicator is very high. 

More recently, the use of genetically engineered fluorescent Ca^2+^-binding peptides (GECIs) has gained importance, since they can report localized Ca^2+^ changes when fused to a suitable cellular protein [21]. Thus far, the modeling of beta cell function is based on observations made with conventional small-molecule indicators, which report the Ca^2+^ concentration in the bulk cytosol. The Ca^2+^ binding affinity plays a major role in the selection of the indicator. Indicators with a high affinity such as Fura 2 [20], arguably the most used conventional Ca^2+^ indicator, tend to underestimate peak values occurring during strong Ca^2+^ influx, and vice versa; low affinity indicators will miss more subtle changes. Finally, the optical properties influence the selection. The Fluo series of indicators [22,23] are best excited at about 490 nm, which makes them useful for laser excitation. In contrast to the ratiometric Fura 2, the Fluo indicators are excited by a single wavelength, which may require the normalization of the data when a mean value of several measurements is calculated. 

### 1.4. Evolution of Models

Mathematical modelling of stimulus–secretion coupling in the pancreatic beta cell began in the early 1980s [24]. However, at that time, the knowledge was too sketchy to permit sufficiently complex models. Only with the further development of electrophysiological and microfluorometric techniques could the oscillation of the membrane potential and of the cytosolic Ca^2+^ concentration during glucose stimulation be adequately characterized [25,26]. The generation of these oscillations and their relevance for the kinetics of insulin secretion and for glucose tolerance have remained a matter of debate ever since. 

Both, the metabolism of glucose and the feedback between cytosolic Ca^2+^ levels and ion channel activity are likely to be relevant [27,28]. For a complete picture, the interactions within the islet have to be taken into account [29]. Correspondingly, the development of models progressed by taking more of the above mechanisms into account. The models are based on coupled non-linear ordinary differential equations to describe the multiplicity of oscillations. Special attention was paid to the fact that the dynamics consist of superimposed oscillations with widely separated period lengths. 

Focusing initially on the electrical phenomena, the “phantom burster model“ was generated [30] based on four state variables. In addition to the membrane potential and the potassium flux, two abstract variables and their time-related constants were included. However, this did not yet allow the description of compound bursting. The next evolutionary step was the “dual oscillator model” (DOM) [31], which was based on seven state variables. Instead of the abstract state variable of the initial model, the corresponding Ca^2+^ concentrations were considered, and three equations described the contribution of the metabolism. In particular, the activity of the glycolytic enzyme phosphofructokinase (PFK) was considered a generator of oscillations. 

A further layer of complexity resulted from the inclusion of ATP binding to the K^+^ channels and ATP activation of the Ca^2+^ pumps of the endoplasmic reticulum (ER) which in turn promotes PFK activity [32]. Here, the activity of PFK was calculated according to the model of [33]. In contrast to previous models, the model presented by [34] contains a direct coupling between the changes of Ca^2+^ and those of ATP. This relation is established via the regulatory effect of Ca^2+^ on the mitochondrial enzyme pyruvate dehydrogenase. Due to the closer relationship between the two origins of oscillation, this model was named the “integrated oscillator model” (IOM). This model incorporated the oscillatory nature of glycolysis as measured in pancreatic beta cells [35]. 

## 2. Results and Discussion

### 2.1. Modeling of Membrane Currents

The ion currents through the plasma membrane play a central role in the modelling. In light of this, it will first be analyzed whether fundamental measurement results can be described with the reference model, and how well the associated parameters meet the experimentally obtained quantities. This appeared necessary, since very little information on the exact method of parameter determination or on the basis of the experiment is contained in the pertaining publications [36,37]. 

**Figure 1:**
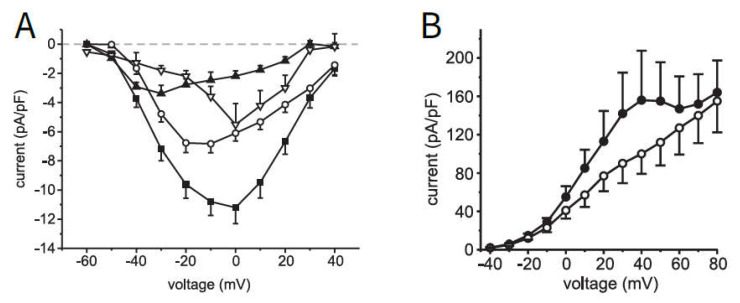
Measurement of current–voltage correlations for Ca^2+^-channels (**A**) and K^+^-channels (**B**); for (**A**): ■ sum of all currents, ◯: L-Type, ▲ P/Q-Type ▽: T-Type; for (**B**): ● current without further agents, ◯: current in the presence of Co^2+^, Reprinted with permission from [38], 2008, American Diabetes Association.

In beta cells, the pattern of action potential spiking results mainly from the currents flowing through the voltage-dependent potassium and Ca^2+^ channels, whereby the transmembrane voltage determines both the open probability of the channels and the driving force of the ion current. To test the descriptive power of the equations and the given set of parameters, the fluxes through potassium and Ca^2+^ channels in human beta cells published by [38] were chosen (see Figure 1).

The Ca^2+^ current (left, A, the total current, marked as ■, is to be considered here) is characterized by the fact that at negative voltages the Ca^2+^ channels are only rarely open ($m_{\infty \;}$ in Table A2, Appendix B is very small), which is why the corresponding current is also small. The probability of the channel being open increases with increasing voltage ($m_{\infty \;}$ becomes larger), but at the same time, the distance to the Ca^2+^ equilibrium potential $V_{Ca}$ decreases, which reduces the driving force and, consequently, the current. 

The outward current through the potassium channels (right, B, the current without the addition of Co^2+^, an inhibitor of Ca^2+^ influx, is marked as ●) is also influenced by the above factors. Since the potassium equilibrium potential is in the negative range, the current increases with increasing voltage. The shoulder is caused by the current through the large-conductance Ca^2+^-activated K channels (BK channels), reacting to the opening of voltage-dependent Ca^2+^ channels. 

**Figure 2:**
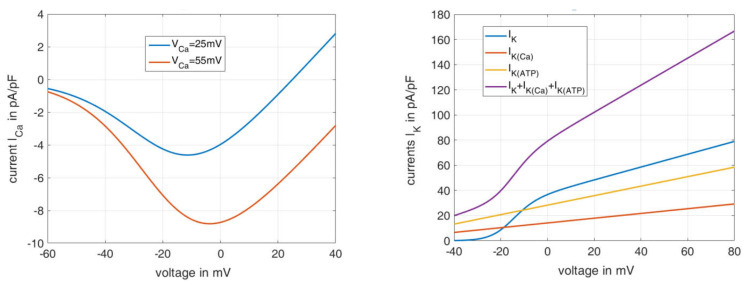
Currents for varying voltages, calculated according to Table A2 (Appendix B), left: current due to calcium ion influx ($I_{Ca}$) for varying Ca^2+^ equilibrium voltages, right: current due to Potassium ion efflux, sum and separated into the components $I_{K},\;I_{K\left(Ca\right)}$ and $I_{K\left(ATP\right)}$.

Figure 2 shows the current–voltage relations for the Ca^2+^ flux (left) and the K^+^ flux (right), as calculated according to the associated formulas in Table A2, Appendix B. The course of the Ca^2+^ current is shown for two different equilibrium Ca^2+^ voltages $V_{Ca}.$ These can take on very different values due to the variability of the cytosolic Ca^2+^ concentration. If a $V_{Ca}$ value of 25 mV is set as in the reference model [36], the formula describes the blue curve, which deviates strongly from the sum of the measured Ca^2+^ currents shown in Figure 1A. If, on the other hand, the $V_{Ca}$ value is set to 55 mV, the resulting orange curve represents the measured values well, both qualitatively and quantitatively. 

The right panel in Figure 2 depicts the curves of the individual potassium ion currents depending on the voltage. For simplification, the steady state is assumed here for $I_{K}$ (i.e.,$\;n=n_{\infty }$), and open channels are assumed for $I_{K\left(Ca\right)}$ and $I_{K\left(ATP\right)}$. The violet curve can be compared with the curves in Figure 1 on the right. The increase between −20 mV and 0 mV can also be seen very well here, and its causation by the opening of the $I_{K}$ channels can be clearly identified (blue curve). A remaining difference is a shoulder with a peak at 40 mV, which does not appear, since the Ca^2+^ influx through voltage-dependent Ca^2+^ channels and its effect on $I_{K\left(Ca\right)}$ were not included in the calculation.

These comparisons between measurements and simulation confirm the potential of the existing equations to represent complex phenomena. On the other hand, it also becomes clear that moderate changes in the parameters can have marked consequences on the system’s behavior.

### 2.2. Inflence of Agents

In this chapter, the reaction of the membrane potential to a set of insulinotropic stimuli is examined. In addition to glucose, which acts as a nutrient secretagogue (see above), the purely depolarizing stimuli, KCl and tolbutamide, are considered. Increasing the extracellular K^+^ concentration raises the potassium equilibrium potential, and tolbutamide reduces the outwardly directed K^+^ current by closing KATP channels. 

#### 2.2.1. Variation of Glucose Concentration 

**Figure 3:**
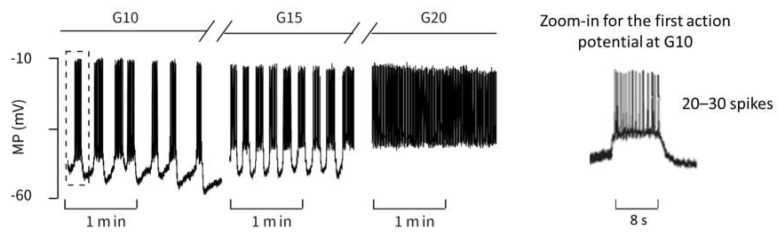
Electric activity of a beta cell within an intact mouse islet in response to glucose, Reprinted with permission from [39], 2014, Elsevier. G10 stands for raising the extracellular glucose concentration from 3 mM to 10 mM, and G15 and G20 for raising to 15 mM and to 20 mM, respectively.

To test how well the model describes the changes of action potential spiking in response to stimulatory glucose concentrations, the measurements published by [39] were chosen. The development of the action potential pattern is depicted in Figure 3. It shows oscillations on different time scales (seconds and hundredths of a second). The high-frequency oscillations represent bursts of action potentials that last a few seconds, separated from each other by electrically silent phases of variable length. At all stimulatory glucose concentrations, the membrane potential oscillates between ca. −45 mV and −10 mV during the action potential bursting, and falls to about −55 mV during the interspersed repolarization phases. The measurements show that raising the glucose concentration to higher values diminishes the distance between the bursting phases until no repolarization occurs at 20 mM glucose. 

To compare this measurement with the simulation, it was purposive to introduce a factor $A_{GC}$ to the ADP-term in the equation describing the relation between ATP and ADP (cf. Equation (A7) in Table A1, Appendix B), since the extracellular glucose concentration is not an explicit component of the model proposed by [36].

(1) $$\frac{dADP}{dt}=\frac{ATP-A_{GC}exp\left[(1+2.2\frac{J_{PDH}}{0.05+J_{PDH}}\right)\left(1-\frac{Ca}{0.35}\right)]ADP}{\tau_{A}}$$

**Figure 4:**
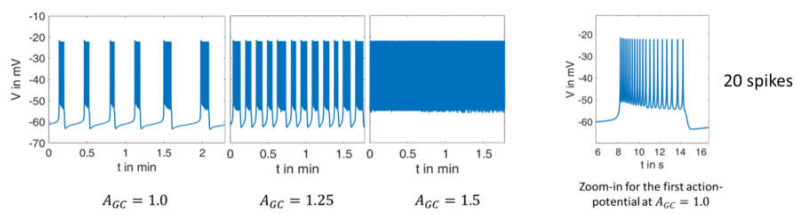
Simulation of the electrical activity as carried out with the model by [36] with varying $A_{GC}$-values.

Actually, in the most recent publication by this group [40] a separate parameter G was included in the equations, but the effect of $A_{GC}$, as used in the present paper, is mathematically comparable. A high value of $A_{GC}$ accelerates the decrease of ADP with increasing extracellular glucose concentrations. With respect to the reference parameter set, the $A_{GC}$ value is set to 1.0 for a glucose concentration of 10 mM, to match the measured curve as closely as possible. Figure 4 shows the calculated courses of the membrane potential and allows a direct comparison with the measurement in Figure 3. The agreement between both is excellent in many respects, and it particularly concerns the following:

- The quantitative values of the membrane potential (except for the small deviations in the maximum potential during the bursts).
- The characteristics of the curves (alternation between high-frequency and low-frequency fractions).
- The length and variability of the phases between the action potentials. In particular, the ability to reproduce this variability with a set of deterministic ordinary differential equations is very remarkable.
- The number of action potentials in the considered time interval.

Zoom-ins of the first action potential in each case are depicted on right-hand panels in Figure 3 and Figure 4. The authors do not have raw data for the measurement from the literature [39] in Figure 3, but from the representation and time scale, it can be read that the duration of this first action potential is approximately 8 s, during which about 20–30 spikes occur. The distance between the spikes is particularly small at the beginning. This property can be compared with the zoom-in from the simulation in Figure 4. Here, the duration of the first action potential is approximately 6 s, in which there are exactly 20 spikes. From this, it can be concluded from the comparison between the measurement and the simulation that

- the duration of the action potentials is of a comparable size.
- the number of spikes in relation to the duration matches very well.
- The distance between the spikes within the action potential increases over time. This is a well-known feature, see, e.g., [41].

The values for $A_{GC}$ that achieve the best possible agreement at higher glucose concentrations were sought for the model, and gave ($G15\to A_{GC}=1.25,\;G20\to A_{GC}=1.5)$. Thus, the relation between the extracellular glucose and the A_GC_value is not linear, which is easily recognizable from the fact that a 50% increase ($A_{GC}=1.5$) results in a depolarization pattern that fits the experimental observations obtained with 20 mM glucose, but not those with 15 mM glucose. If one now compares simulation and measurement from the point of view of varying glucose supply, the following conclusions can be drawn:

- In the simulation as well as in the measurement, the time between two bursts of action potentials shortens with increasing $A_{GC}$ (increasing glucose supply).
- At G20 or $A_{GC}=1.5$, respectively, there are practically no more resting phases; action potentials are permanently present.
- In the experimental registrations, the peak value of the action potentials decreases with increasing G and time; in the simulation, this value remains nearly constant (at about −20 mV).

**Figure 5:**
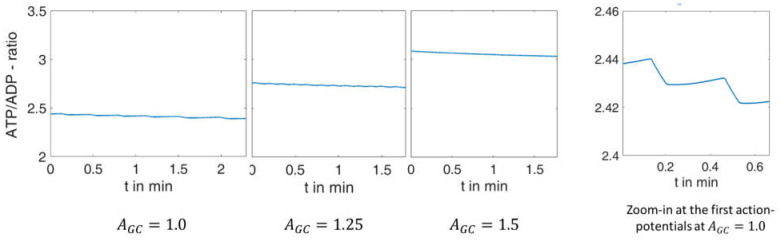
ATP-ADP ratio over time for the processes simulated in Figure 4.

As a further characteristic, the ratio of ATP/ADP can be specified for these systems at each point in time. The corresponding curve is shown in Figure 5.

This figure shows that the model modification described above couples the ATP/ADP ratio to the increasing extracellular glucose concentration, a relation that has repeatedly been demonstrated experimentally [42,43]. Primarily, this feature affects the mean value. During the electrical activity, a decrease in the ATP/ADP ratio does occur over time (best seen in the zoom-in to the right), but this is orders of magnitude smaller than that due to a decrease in glucose concentration. 

In order to test the robustness of the model, the values of one of the parameters were changed. In beta cells, the influx of Ca^2+^ forms the upstroke of the action potential [44]. At a given open probability of the Ca^2+^ channels, the flux is proportional to the difference between the actual membrane potential, which is typically in the negative range, and the Ca^2+^ equilibrium potential ($V_{Ca}$), which is in the positive range. Consequently, the larger the value of $V_{Ca}$ is, the larger the flux will be. $V_{Ca}$ was only moderately changed; it decreased from 25 mV to 20 mV and increased from 25 mV to 30 mV. 

**Figure 6:**
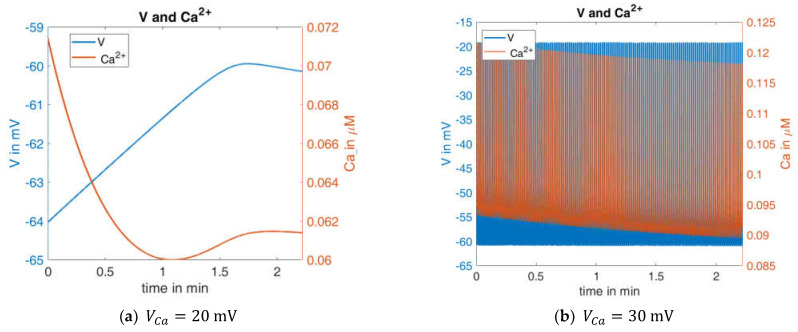
Effect of a slight change in resting Ca^2+^ potential; simulated curves for the membrane potential (blue) and Ca^2+^ concentration (orange) for (with respect to the reference configuration) (**a**) a slight decrease in $V_{Ca}$ (**b**) a slight increase in $V_{Ca}$.

In Figure 6, left panel shows the curves of V and Ca for the first interval (G10, $A_{GC}=1$). The blue curve can be directly compared with the left curve in Figure 4. When $V_{Ca}$ is reduced to 20 mV, no action potentials occur. The membrane potential and the cytosolic Ca^2+^ concentration change only marginally over time, and then reach a steady state. In the case of an increased $V_{Ca}$ value (30 mV, Figure 6, right panel), the behavior is virtually the opposite; continuous large-amplitude action potentials are accompanied by a high-frequency oscillation of the cytosolic Ca^2+^ concentration. 

This extreme reaction to a moderate change of one model parameter is an indication of how much the informative value of this model depends on the right choice of the model parameters. Since it cannot be assumed that a real beta cell has a similar sensitivity to the Ca^2+^ equilibrium potential, a further evolution of the model seems necessary to improve its robustness.

#### 2.2.2. Variation of K^+^ Concentration 

**Figure 7:**
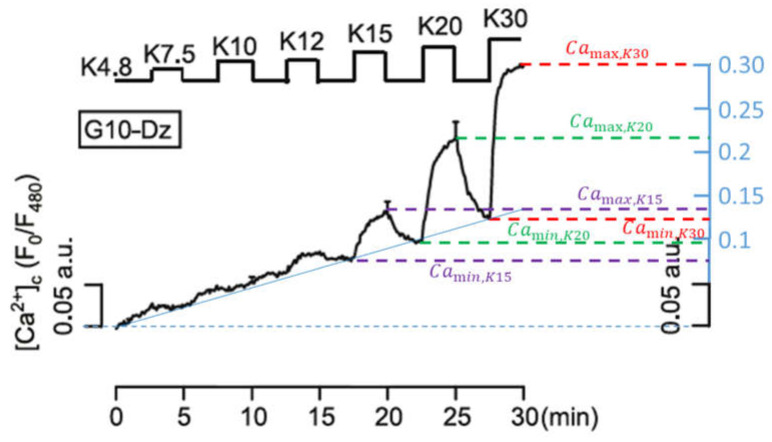
Dynamics of the cytosolic Ca^2+^ concentration under different given extracellular potassium concentrations in the presence of 10 mM glucose and 100 μM diazoxide. , Reprinted with permission from [39], 2014, Elsevier.

Raising the extracellular K^+^ concentration from a physiological value of about 5 mM to 30 or 40 mM, or even higher, is a widely used experimental procedure to elicit a Ca^2+^ influx via voltage-dependent Ca^2+^ channels. The resultant increase of the cytosolic Ca^2+^ is sufficient to stimulate the secretion of insulin [45]. To test the ability of the model to correctly describe a complex pattern of the cytosolic Ca^2+^ concentration in response to changes in the extracellular K^+^ concentration, we chose the measurement published by [39] and depicted in Figure 7. Here, the potassium concentration is increased stepwise from 7.5 mM to 30 mM, interrupted by 2.5 min intervals at the physiological value of 4.8 mM. The authors of the present paper have added the blue scale on the right of the figure to further quantify the results, in order to be able to compare individual quantities with the simulations.

Up to the increase in the potassium concentrations to 12 mM, a nearly continuous (linear over time, see solid blue line) increase of the cytosolic Ca^2+^ concentration predominates until a self-reinforcing effect occurs due to progressively stronger depolarisation, which causes the Ca^2+^ concentration to rise rapidly before it decreases again rapidly with a reduction in the potassium concentration. The difference between the temporary maxima (right before the potassium concentration is reduced again) and the temporary minima (immediately before the potassium concentration is increased again), increases with increasing potassium concentrations. These are clearly readable, especially in the last three oscillations (cf. Figure 7), and are listed in Table 1:

For the model, the influence of the changing potassium concentration is taken into account via the potassium equilibrium potential $V_{K}$ . For this purpose, the value of $V_{K}$ is changed abruptly for the respective time intervals, using the Nernst equation as a basis, and assuming a temperature of T=310 K and an intracellular potassium concentration of $K_{intra}=130\;\mathrm{mM}$:

(2) $$V_{K}=R\frac{T}{F}ln\left(\frac{K_{extra}}{K_{intra}}\right)=8.314\;\frac{{\mathrm{kg}\;m}^{2}}{s^{2}\;\mathrm{mol}\;K}\frac{310K}{96485.332\frac{C}{\mathrm{mol}}}ln\left(\frac{K_{extra}}{130\;\mathrm{mM}}\right)=26.7\times 10^{-3}V\;ln\left(\frac{K_{extra}}{130\;\mathrm{mM}}\right)$$

**Figure 8:**
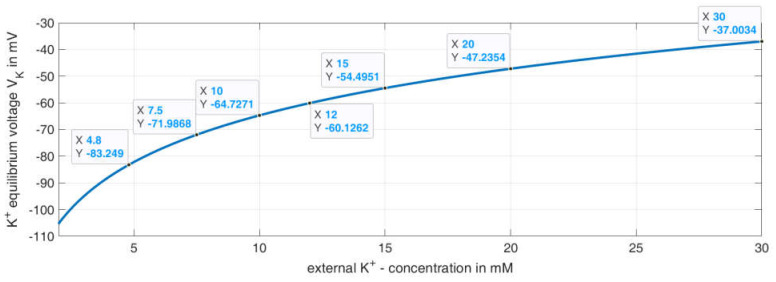
Course of the K^+^ equilibrium potential over the external potassium concentration and data points for the values used in the model.

This formula generated the curve for $V_{K}$ depending on the extracellular K+ concentration, as shown in Figure 8. The data tips represent the values used for the given K^+^ concentrations in the model.

**Figure 9:**
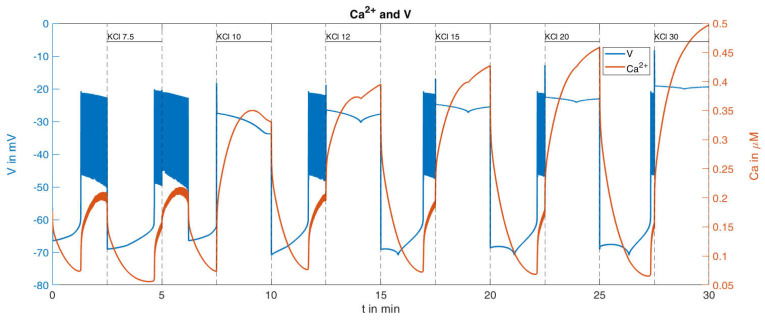
Dynamics of Ca^2+^ concentration and corresponding membrane potential under different given extracellular potassium concentrations; reference curve calculated with the model from [36].

With this procedure and the parameters set from the reference [36], the response of the cytosolic Ca^2+^ concentration and of the plasma membrane potential was simulated (Figure 9). With respect to the Ca^2+^ values (orange curve in Figure 9), certain similarities within the measurements can be seen, such as the increasing rise of the peaks for increasing K^+^ concentrations; however, clear differences are also visible, such as the Ca^2+^ increases at the low K^+^ concentrations. In particular, the bursts of action potentials (blue curve in Figure 9), occurring even before the K^+^ concentration was raised, are a cause for concern. Since the potassium channels remain open during K^+^ depolarization, no action potentials are generated by the beta cells [46]. In the given example, the open state of the KATP channels, which make up the major part of the K^+^ conductance, was ensured by the presence of diazoxide. 

**Figure 10:**
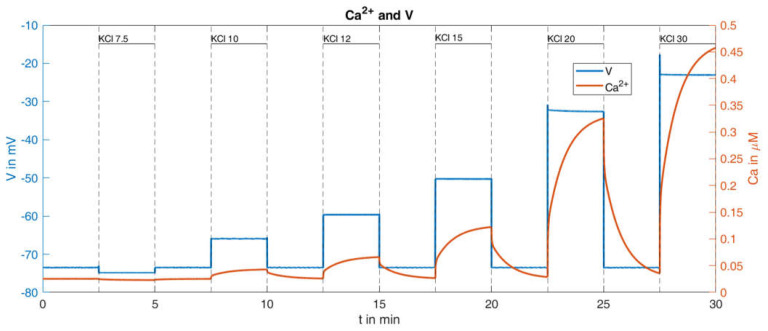
Dynamics of Ca^2+^ concentration and corresponding membrane potential under different given extracellular potassium concentrations, calculated with a higher value of $o_{\infty }(o_{\infty })=0.03$.

The consideration of this influence was not a major purpose of the reference model [36] and was therefore not taken into account. In the model, the permeability of the KATP channels is essentially determined by the parameter $o_{\infty }$, compare Table A2 in Appendix B. In the simulation described above, this value fluctuates slightly around a value of about 0.006. In order to adequately incorporate the influence of diazoxide, the parameter was subsequently set to the constant value of $o_{\infty }=0.03$, i.e., about five times as large as the reference value. This means that the KATP channels are significantly more permeable than in the calculated reference configuration. Keeping all other parameters unchanged, the curves shown in Figure 10 are obtained.

This change of parameter results in the absence of action potentials, and the rise of the cytosolic Ca^2+^ concentration shows greater similarity with the measurement. The quantitative comparison between the simulation results and the measurement is presented in Table 2:

It turns out that this relatively small change in system parameters matches the simulation much better to the measured behavior than the original set of parameters. 

**Figure 11:**
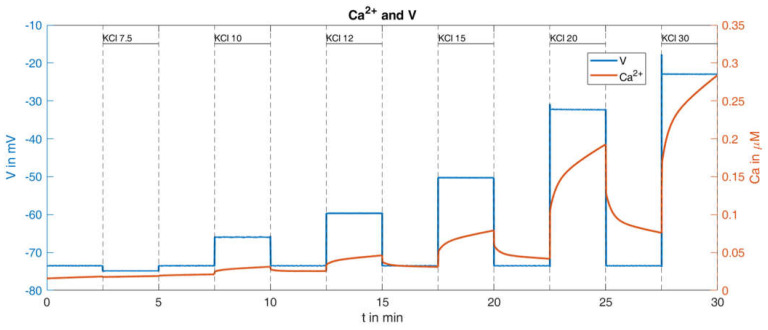
Dynamics of Ca^2+^ concentration and corresponding membrane potential under different given extracellular potassium concentrations, calculated with a higher value of $o_{\infty }(o_{\infty })=0.03$ and highly reduced (1/10 of the reference values) intracellular Ca^2+^ transport parameters ($p_{leak}=2\times 10^{-5}{\;\mathrm{ms}}^{-1}$ and $k_{NaCa}=1\times 10^{-4}{\;\mathrm{ms}}^{-1}$ ).

The remaining difference between the simulated Ca^2+^ response and the measured Ca^2+^ response is largely caused by the lacking linear increase of the minimal values between the K^+^ depolarizations. This was addressed by decreasing the terms describing the exchange of Ca^2+^ between the ER and the cytosol and between the mitochondria and the cytosol (see Equation (A4) in Table A1, Appendix B). While a decrease to 50% resulted in a very modest increase, a decrease to 10% generated a clearly visible increase, see Figure 11.

In conclusion, the model as such is very able to reproduce kinetic responses to a mechanistically simple stimulation with considerable robustness.

#### 2.2.3. Effects of Pharmacological Block of KATP Channels by Tolbutamide 

**Figure 12:**
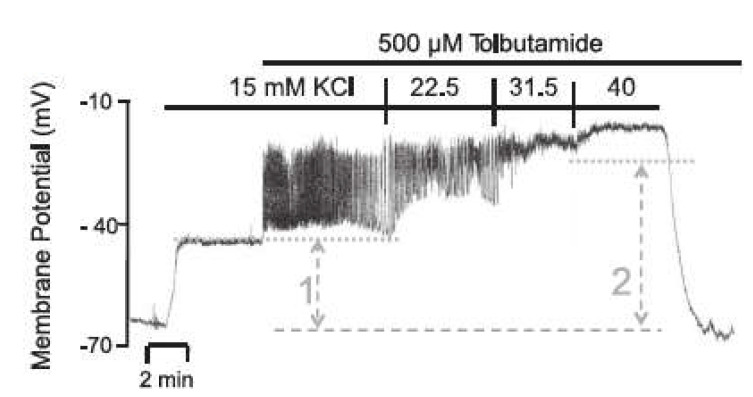
Membrane potential of a pancreatic beta cell in the presence of 500 µM tolbutamide and stepwise increases of the potassium concentration (Reprinted with permission from [47], 2014, the American Physiological Society).

In contrast to the depolarization maneuver by high extracellular K^+^ concentration, the block of KATP channels produces a depolarization of the beta cell with action potential spiking. A virtually complete closure of the channels can be achieved by 500 μM of the sulfonylurea, tolbutamide. Interestingly, the amplitude of the action potentials diminishes to virtual non-existence when the extracellular K^+^ concentration is raised from 15 mM to 40 mM [47], see Figure 12.

The addition of 15 mM KCl leads to a moderate depolarization of the membrane potential (comparable to the behavior in Section 2.2.2). Only after the addition of tolbutamide and the resulting blockade of the KATP channels does a rapid depolarization of the membrane potential occur, as the depolarizing Ca^2+^ influx can no longer be balanced by a repolarizing K^+^ efflux. The additional depolarization opens the potassium channels through which the current $I_{K}$ now leads to potassium efflux, which decreases the membrane potential before Ca^2+^ influx again predominates and the membrane potential rises again. With the subsequent increase in the extracellular K^+^ concentration, the K^+^ equilibrium potential $V_{K}$ is increased, whereby all repolarizing K^+^ currents are diminished, inhibiting the generation of action potentials. 

The simulation accounts for the addition of tolbutamide in the way that, according to Hill’s equation, the parameter $g_{K\left(ATP\right)}$ is modified, which considers the permeability of the ATP-dependent potassium channels. Specifically, in this model extension, the tolbutamide concentration T determines the tolbutamide-dependent permeability according to Equation (3):

(3) $$g_{K\left(ATP\right),Tolb}=g_{K\left(ATP\right)},\left(1-\frac{T^{n}}{T_{0.5}^{n}+T^{n}}\right)$$

with the half-effect concentration of $T_{0.5}^{n}=1.90\;µM$ [48] and the HILL exponent n=1, it can be obtained for the case T=500 µM: $g_{K\left(ATP\right),Tolb}=2.69\times 10^{-3}g_{K\left(ATP\right)}$, which corresponds to a very low residual permeability.

**Figure 13:**
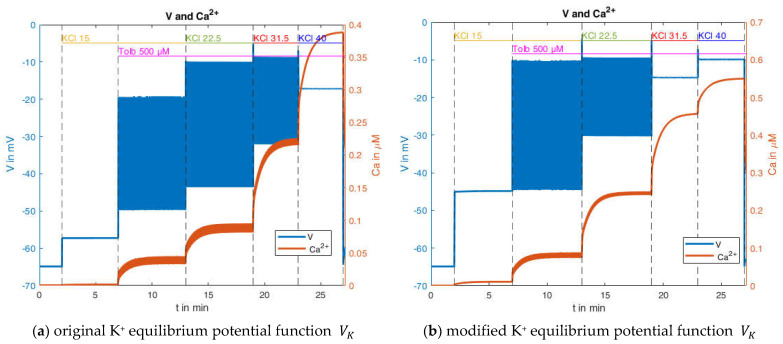
Simulated membrane potential and cytosolic Ca^2+^ concentration in the presence of 500 µM tolbutamide and stepwise increases of the potassium concentration, considering (**a**) the original equilibrium potential function (Equation (2)), and (**b**) a modified equilibrium potential function (10 mV larger than the original).

With the slight modification of $u_{m}=-15\;\mathrm{mV}$ and $s_{m}=6\;\mathrm{mV}$ (without these there would be no bursts) the behavior depicted in Figure 13a can be generated. It bears considerable resemblance to the experimental measurement shown in Figure 12. There are no bursts in the presence of 15 mM KCl, but the membrane potential is already elevated; however, the value of about −57 mV is still significantly below that of the measurement. With the addition of tolbutamide, the action potentials become visible. With a further increase of the KCl concentration to 22.5 mM, the mean membrane potential continues to rise, but the amplitude of the action potentials increases too, which does not conform with the measurement. With the increase to 30 mM KCl, the mean membrane potential continues to increase, but the amplitude of the oscillation decreases, which is closer to the characteristic of the measurement. Finally, at 40 mM KCl, the bursting ends, as in the measurement.

For these calculations, the equilibrium potentials according to Equation (2) have been used, which exclusively considers potassium ions. While the potassium conductance is by far dominating at the resting state, other conductances through voltage-dependent channels play a role during prolonged depolarization. To account for this influence phenomenologically and to get first ideas on this effect, another simulation was performed, wherein the potential calculated according to Equation (2) was artificially increased by 10 mV. The curves resulting from this modification are shown in Figure 13b. The simulated membrane potential curve now closely resembles the one in the measurement. The membrane potential before the addition of tolbutamide is −45 mV, and the mean membrane potential during action potential spiking agrees better with the measurement. Furthermore, the marked decrease in action potential amplitude is now also evident when the K^+^ concentration is increased from 15 mM to 22.5 mM.

### 2.3. Influence of the Measurement Itself 

As mentioned in Section 1.3, the cytosolic Ca^2+^ concentration is usually measured by fluorescent indicators, whereas the plasma membrane potential is best measured by electrophysiological techniques. Thus, it is possible to combine the measurement of the membrane potential with the microfluorimetric measurement of the cytosolic Ca^2+^ concentration. Due to the fact that the occurring voltages and currents are very small, there are high demands on the measurement system itself, especially to exclude a feedback effect of the measurement on the system behavior. 

**Figure 14:**
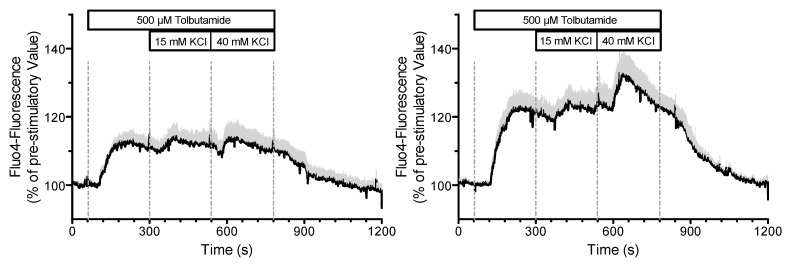
Measurement of the cytosolic Ca^2+^ concentration of a pancreatic beta cell in the presence of 500 µM tolbutamide and stepwise increases of the potassium concentration; left: with simultaneous current clamp measurement; right: with the patch pipette sealed on the cell, but without applied voltage. Data are means ± SEM of four experiments.

An indication that there may be a feedback effect of the measurement on the system is shown in Figure 14. In this experiment, the K^+^ concentration was increased stepwise in the presence of tolbutamide, first to 15 mM, then to 40 mM. In the left panel, the plasma membrane potential was simultaneously measured by current clamping in the “perforated patch” configuration. In the right panel, the same configuration was generated, but no current clamp was applied. 

It is obvious that the Fluo 4-fluorescence and thus the cytosolic Ca^2+^ concentration is significantly smaller with the simultaneous measurement of the membrane potential than that without. If the measurements are feedback free, there should be no difference between the measurements.

**Figure 15:**
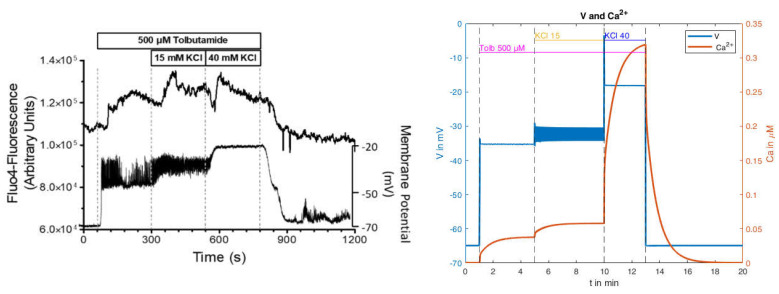
Simultaneous measurement of the membrane potential and the cytosolic Ca^2+^ concentration of a pancreatic beta cell in the presence of 500 µM tolbutamide and stepwise increases of the potassium concentration; (**left**): original measurement; (**right**): simulated values.

In the following, we attempt to derive an explanation for this effect, based on the model presented. For this purpose, a single measurement from the mean values in the left panel of Figure 14 will be used first (Figure 15, left panel).

The calculated curves for this experiment as obtained with the parameters from Table A3 (in Appendix B) are shown in Figure 15 on the right. The depolarization pattern is approximately well matched, and the voltage is in the correct range. However, no action potentials are generated by the addition of tolbutamide, whereas the transition from 15 mM to 40 mM KCl abolishes the preexistent action potentials, conforming with the measurements. For the cytosolic Ca^2+^ concentration, a moderate increase comparable to the measurement occurs with both the addition of tolbutamide and the subsequent KCl addition. In contrast to the measurement, however, the brief Ca^2+^ decrease after the addition of 40 mM KCl is missing, and the subsequent increase is much stronger in the model than in the measurement. This is still true when taking into consideration that the simulation gives cytosolic Ca^2+^ values, whereas the measurement shows that Fluo-4 fluorescence under-reports large increases due to its highly non-linear correlation. To illustrate this point, it is remarkable that the peak value of the Fluo-4 fluorescence in the presence of 15 mM KCl is virtually the same as the one in the presence of 40 mM KCl. Of note, in the control experiments, the peak value at 40 mM KCl was significantly higher than the one at 15 mM KCl (Figure 15, left panel). 

Thus, the simulation can reproduce the features of this experiment in a first approximation. So, it was interesting to test whether it could be used to investigate the possible reasons as to why the current clamp mode affects simultaneously measured cytosolic Ca^2+^ concentrations. 

**Figure 16:**
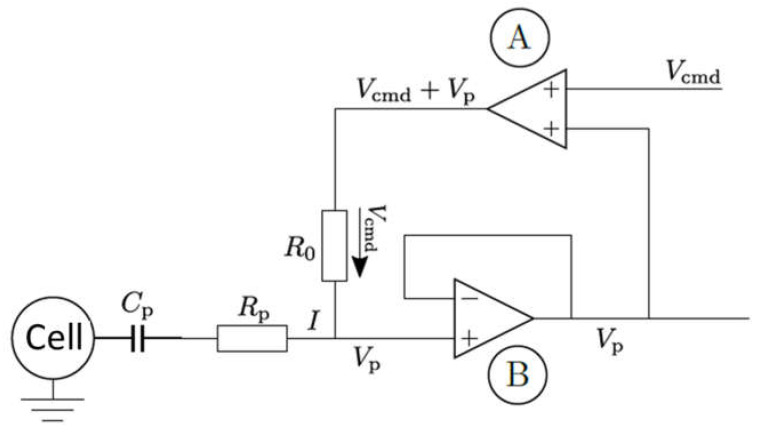
Principle sketch for patch clamp measurements.

In order to understand the possible mechanisms, detailed information about the electrical circuit of the measuring apparatus is necessary. A principle schematic is shown in Figure 16 [49]. In addition to the operational amplifiers and impedance converters, which provide the voltages that cause the specified current, the main electrical components are the resistance of the pipette and its capacitance. For the simulation, both the resistance and the capacitance of the pipette are to be understood as a series connection to the resistance and the capacitance of the cell membrane (compare Figure A1). Resistances connected in series are added. The resistance of the pipette is much smaller than the resistance of the cell membrane, and can therefore be neglected.

The situation is rather different with the capacitances. The capacitance of the pipette $C_{p}$ is in the range of 1–5 pF [49] and is thus in the order of magnitude of the capacitance of the cell (5.3 pF was assumed in the model, see Appendix B). A series connection of two capacitances is made by adding the reciprocal values.

The effective capacity is therefore

(4) $$\frac{1}{C_{res}}=\frac{1}{C_{m}}+\frac{1}{C_{p}}\to C_{res}=\frac{C_{m}C_{p}}{C_{m}+C_{p}}$$

Depending on whether one now assumes the minimum, maximum, or an average value of 3 pF for the pipette capacitance, this results in $C_{res,max}=2.57\;\mathrm{pF}$, $C_{res,min}=0.84\;\mathrm{pF}$ and $C_{res,mean}=1.92\;\mathrm{pF}$.

**Figure 17:**
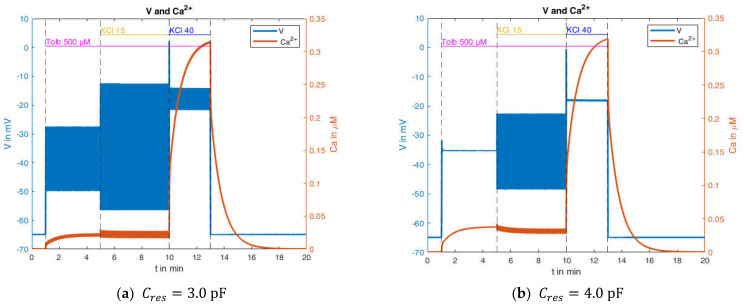
Membrane potential and Ca^2+^ concentration under the addition of 500 µM tolbutamide and varying potassium concentrations with reduced resulting capacities.

In any case, the resulting capacitance is significantly below the membrane capacitance. In order to investigate this influence, tests were subsequently carried out on the influence of the “true” capacitance (see Equation (4)). The model showed instabilities at very small capacitances, which is why moderate changes in capacitance are considered below. In this context, the original value of 5.3 pF was set to the values 3.0 pF and 4.0 pF, respectively. The corresponding results are shown in Figure 17.

Comparing these curves with the simulation resulting from the use of the original capacitance value (Figure 15, right) reveals clear differences. The reduction of the capacitance results in a higher propensity of the system to produce action potentials, and at the same time, in a diminished increase of the cytosolic Ca^2+^ concentration. When no action potentials or action potentials with a small amplitude are present, the Ca^2+^ concentration approaches the same steady-state value as in the original simulations. This is also the reason that the response of the Ca^2+^ concentration to the transition from 15 mM to 40 mM KCl is nearly unchanged.

In conclusion, the measurement of the plasma membrane potential by current clamping appears to affect the simultaneous measurement of the cytosolic Ca^2+^ concentration because of the reduced effective capacitance of the system. This effect is the likely explanation for the observations depicted in Figure 14. Bearing this in mind, a suitable model should be able to subsequently calculate the effect of the measurement and to compensate for the feedback effect of the measurement system. 

## 3. Materials and Methods

### 3.1. Chemicals

Collagenase NB8 (Serva, Heidelberg, Germany) and collagenase P from Roche (Sigma-Aldrich, Deisenhofen, Germany) were used for islet isolation. Tolbutamide was obtained from Sigma, and Fluo 4 from Thermo-Fisher Scientific (Schwerte, Germany). Cell culture medium RPMI 1640 (without glucose) was from Sigma, and fetal calf serum (FCS Gold ADD) was from Bio & Sell (Nürnberg-Feucht, Germany). All other reagents of analytical grade were from E. Merck (Darmstadt, Germany). 

### 3.2. Tissue and Tissue Culture

Islets were isolated from the pancreas of NMRI mice (12–16 weeks old, fed ad libitum) by a collagenase digestion technique and hand-picked under a stereomicroscope. Single islet cells were obtained by incubation of the islets for 10 min in a Ca^2+^-free Krebs-Ringer medium and vortex mixing. Single cells were cultured in a humidified atmosphere of 95% air and 5% CO_2_ at 37 °C for 24 h in RPMI containing 10% FCS. The glucose concentration was 10 mM for the first 2 h, then 5 mM. Animal care is supervised by the regional authority (LAVES, Lower Saxony, Germany) and conforms to the current EU regulations.

### 3.3. Measurement of the Plasma Membrane Potential 

The plasma membrane potential of 1-day- or 2-day-cultured single islet cells was measured using the patch clamp technique in the perforated patch configuration. Pipettes were pulled from borosilicate glass (2 mm o.d., 1.4 mm i.d., Hilgenberg, Germany) by a two-stage vertical puller (HEKA-Electronics, Lambrecht, Germany) and had resistances between 3 and 6 MΩ when filled with solution. The measurements were performed using an EPC 7 patch clamp amplifier (HEKA-Electronics) in the current clamp mode and pClamp 6.03 software (Axon Instruments, Foster City, CA, USA). The data were stored on a hard disk and analysed offline using GraphPad Prism5 software (GraphPad, LaJolla, CA, USA). A slow bath perfusion system was used, and all experiments were performed at room temperature (20–22 °C). The pipette solution contained the following (mM): 10 KCl, 10 NaCl, 70 K_2_SO_4_, 7 MgCl_2_, and 5 HEPES, pH 7.15, plus 125 µg/mL of the pore-forming agent nystatine (2.5% DMSO final concentration). The extracellular solution contained (mM) 140 NaCl, 5.6 KCl, 1.2 MgCl_2_, 2.6 CaCl_2_, 10 HEPES, and 1 glucose, pH 7.4. 

### 3.4. Microfluorimetric Measurement of the Cytosolic Ca^2+^ Concentration 

During the measurement of the plasma membrane potential, the cytosolic Ca^2+^ concentration of single islet cells was measured using Fluo 4 (loaded at a concentration of 1 µM for 20 min at 37 °C). Excitation was at 490 nm, the emission (>510 nm) was collected with a Leitz fluorite objective (40×, 1.3 N.A.) and registered by a photon-counting multiplier (Hamamatsu H6240-01) at 2 Hz under the control of LabView 7.1 (National Instruments, Munich, Germany). 

### 3.5. Modelling

**Figure 18:**
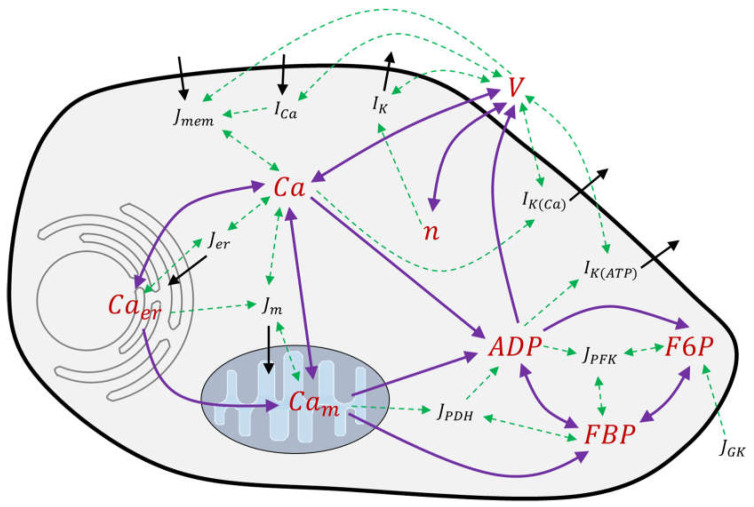
Interactions considered mathematically in the integrated oscillator model—red: eight state variables; black: associated flows, transport and reaction kinetics; green: explicit mathematical interactions; violet: mathematical coupling between the state variables. The direction of the arrow indicates which state variable generates immediate input into the dynamics of the respective other state variables. The state variables interact with each other via currents, transports and reaction kinetics (each in blackletters). The associated equations, measures, and parameter values are listed in Table A1, Table A2 and Table A3 in Appendix B.

The advanced state of modeling, as represented by the “integrated oscillator model” (IOM), was chosen to test whether it has achieved general applicability to describe the changes observed in single beta cells (Figure 18) by independent experimentation. The number of eight state variables in this model (Table 3) permitted us to model various modes of bursting, which include fast bursting, slow bursting, and compound bursting [36,37]. The corresponding equations and parameters are listed in Table A1, Table A2 and Table A3 in Appendix B. All the following numerical studies have been carried out with the tool MATLAB R 2020b. Specifically, the present study aims to answer the following questions:

1. How robust is the model when tested considering novel experimental results?
2. How good is the response of the model to pharmacological agents?
3. Is the model helpful to understand the influence of the measuring technique?

The measurements, in which various agents are applied, are always designed in such a way that they start from a steady state. In order to be able to compare the measurement results with the simulation results, it is therefore very important to also start from the steady state in the simulations. This is particularly important because, in this highly non-linear system, there are dynamics on different time scales that can significantly influence the system. For this reason, a simulation was carried out for each simulation before the actual time integration, in which practically the agent-free state is characterised and in which a steady state is calculated over sufficiently long simulation times. This state forms the initial condition for the actual simulations. The numerical time integration has been implemented using the conventional MATLAB intern integrator ode45.

## 4. Conclusions and Outlook

In the present paper, the model of Marinelli et al., 2018 [36], used to describe the events of stimulus secretion-coupling within the pancreatic beta cell, was tested using published and unpublished measurements of the plasma membrane potential and the cytosolic Ca^2+^ concentration. We confirm that the model is well suited to represent the dynamics and interactions of membrane potential, Ca^2+^ transport, and the metabolism of glucose. Furthermore, the usefulness of simulating experimental results h shown by investigating the unexpected observation that measuring the membrane potential affects the simultaneous measurement of the cytosolic Ca^2+^ concentration. 

However, it was also found that the model is very sensitive to slight variations in some parameters, and may have to be adjusted differently to correctly describe a wider range of observations obtained by other groups. A high degree of sensitivity is in particular due to the description of the permeability of the different ion channels. For example, in the case of the ATP-dependent potassium channels, there is a close interaction between tolbutamide and ADP or ATP that has not yet been taken into account in the model. 

A similar example of yet-to-be-considered complexity is given by direct interactions between intracellular Ca^2+^ stores, such as those recently described by Klec et al. for a mitochondrially directed Ca^2+^ leak of the ER in beta cells [50,51]. So far, the interactions considered mathematically are based on the assumption that Ca_er_ and Ca_m_ are only linked via Ca, the free Ca^2+^ concentration in the bulk cytosol which equally affects processes at the plasma membrane. Therefore, this particular effect is not included in the model’s present stage of evolution, but can be considered in future works.

To achieve a generally valid description of the stimulus–secretion coupling within the beta cell, it seems desirable to include the effects of non-glucidic nutrient secretagogues such as alpha-ketoisocaproic acid, or the combination of glutamine with leucine. Since the effects of these stimuli include membrane depolarization and Ca^2+^ influx, their integration should be possible within the current conceptual framework. Another desirable feature to include in the model is the physiologically relevant endpoint, which is, after all, insulin secretion (see, e.g., the modeling of insulin secretion as a function of the actin cytoskeleton, [52]).

To achieve this goal, two further aspects have to be considered. First, Ca^2+^ microdomains in the vicinity of the Ca^2+^ channels are considered to be the immediate fusion signal, and may not be well represented in the bulk cytosolic Ca^2+^ concentration [53,54]. Based on recent work by Felix-Martinez et al. [55], spatially resolved calcium concentrations can also be calculated, and lead to very significant gradients within the cytosol (they are also correlated with insulin secretion models [56]). For future studies, this would also raise questions about the assumption of a homogenised consideration of the Ca^2+^ in the system of equations discussed here. Corresponding model extensions could be used as a possible approach to address this issue. 

Second, the efficiency of the Ca^2+^ signal is modulated by the amplifying signals [6,7] which have thus far eluded unequivocal identification. So, the task of modeling the stimulus–secretion coupling of the pancreatic beta cell offers a large bandwidth of opportunities for further work. 

## Figures and Tables

**Table 1:** Characteristic values for the Ca^2+^ concentration in arbitrary units, as derived from Figure 7.

	$Ca_{max}\;$	$Ca_{min}$	$D=Ca_{max}-Ca_{min}$	$D_{K30}/D_{K,i}$
K30	0.30	0.12	0.18	1
K20	0.22	0.09	0.13	1.38
K15	0.14	0.08	0.06	3.0

**Table 2:** Characteristic values for the simulated Ca^2+^ concentration and comparison to the measurement in [39].

	$\mathrm{Ref}.\;\mathrm{Simulation}\;o_{\infty }\approx 0.006$		$\mathrm{Simulation}\;\mathrm{with}\;o_{\infty }=0.03$		Measurement
	$Ca_{max}$	$Ca_{max,K30}/Ca_{max,K,i}$	$Ca_{max}$	$Ca_{max,K30}/Ca_{max,K,i}$	$D_{K30}/D_{K,i}$
K30	0.50	1	0.46	1	1
K20	0.46	1.09	0.33	1.39	1.38
K15	0.43	1.16	0.13	3.54	3.0

**Table 3:** Eight state variables of the integrated oscillator model and their meaning.

State Variable	Meaning
V	Membrane potential
n	Potassium ion activity
Ca	Calcium ion concentration in the cytosol
$Ca_{m}$	Calcium ion concentration in the mitochondria
$Ca_{er}$	Calcium ion concentration in the endoplasmic reticulum
F6P	Amount of fructose-6-phosphate
FBP	Amount of fructose-6-biphosphate
ADP	Amount of ADP

## Data Availability

The data obtained in this study, including the MATLAB program, are available upon reasonable request.

## Appendix A. Simplified Electrical Model for the Cell Membrane

The electrical events at the plasma membrane can be considered as taking place in the simplified circuit (see Figure A1) to allow further calculations. The standard model represents the membrane by its electrical resistance, capacity, and resting potential. In contrast to technical circuits, the charge is not transported by electrons flowing through a conductor, but by ions. These can have different charges, such as, e.g., calcium ions with their double positive charge [57].

In this model, the total current flow $I_{m}$ is composed of the capacitive flow $I_{C}$ and the ionic current $I_{i}$. is mainly determined by the specific membrane capacitance $C_{m}$. This depends primarily on the cell type and is almost constant. The ion current depends on the resistance of the membrane $R_{m}$, which is dependent on the transmembrane voltage and the Nernst potential $E_{r}$, which is determined by the ion concentration on both sides of the membrane. For this circuit, this results in the following equation for the current $I_{m}$:

(A1) $$I_{m}=I_{C}+I_{i}=C_{m}\frac{dV_{m}}{dt}+\frac{V_{m}-E_{r}}{R_{m}}$$

The total ion current is composed of different ion currents, such as the flow of sodium, potassium, calcium, or chloride ions.

## Appendix B. Governing Equations and Parameters

**Table A1:** The concrete equations as set up in [36].

$\frac{dV}{dt}=-\frac{1}{Cm}\left(I_{Ca}+I_{K}+I_{K\left(Ca\right)}+I_{K\left(ATP\right)}\right)$	(A2)
$\frac{dn}{dt}=\frac{n_{\infty }\left(V\right)-n}{\tau_{n}}$	(A3)
$\frac{dCa}{dt}=f_{Ca}\left(J_{mem}-J_{m}-J_{er}\right)$	(A4)
$\frac{dCa_{m}}{dt}=f_{Ca}\sigma_{m}J_{m}$	(A5)
$\frac{dCa_{er}}{dt}=f_{Ca}\sigma_{er}J_{er}$	(A6)
$\frac{dADP}{dt}=\frac{ATP-exp\left(1+2.2\frac{J_{PDH}}{0.05+J_{PDH}}\right)\left(1-\frac{Ca}{0.35}\right)ADP}{\tau_{A}}$	(A7)
$\frac{dFBP}{dt}=J_{PFK}-\frac{1}{2}J_{PDH}$	(A8)
$\frac{dF6P}{dt}=0.3(J_{GK}-J_{PFK})$	(A9)

**Table A2:** Measures, additional formulae and meaning of terms related to the integrated oscillator model, taken from [36].

Measure and Formula	Meaning
$I_{Ca}=g_{Ca}m_{\infty }\left(V\right)\left(V-V_{Ca}\right)$	Current due to Calcium ion influx
$m_{\infty }\left(V\right)={\left(1+\exp(\left(u_{m}-V\right)/s_{m})\right)}^{-1}$	$\mathrm{Degree}\;\mathrm{of}\;\mathrm{opening}\;\mathrm{of}\;\mathrm{the}\;{\mathrm{Ca}}^{2+}\;\mathrm{channels}$
$I_{K}=g_{K}n\left(V-V_{K}\right)$	Current due to Potassium ion efflux
$I_{K\left(Ca\right)}=g_{K\left(Ca\right)}q_{\infty }\left(Ca\right)\left(V-V_{K}\right)$	Current due to Calcium-dependent potassium ion efflux
$q_{\infty }\left(Ca\right)=Ca^{2}/\left(k_{d}^{2}+Ca^{2}\right)$	$\mathrm{Degree}\;\mathrm{of}\;\mathrm{opening}\;\mathrm{of}\;\mathrm{the}\;\mathrm{Calcium}-\mathrm{dependent}\;K^{+}\mathrm{channels}$
$I_{K\left(ATP\right)}=g_{K\left(ATP\right)}o_{\infty }\left(ADP,ATP\right)\left(V-V_{K}\right)$	Current due to ATP-dependent potassium ion efflux
$o_{\infty }\left(ADP,ATP\right)=\frac{0.08+0.89{\left(\frac{MgADP}{k_{dd}}\right)}^{2}+0.16\left(\frac{MgADP}{k_{dd}}\right)}{{\left(1+\frac{MgADP}{k_{dd}}\right)}^{2}\left(1+\frac{ATP^{4-}}{k_{tt}}+\frac{ADP^{3-}}{k_{td}}\right)}$	$\mathrm{ATP}-\mathrm{dependent}\;\mathrm{degree}\;\mathrm{of}\;\mathrm{opening}\;\mathrm{of}\;K_{ATP}\mathrm{channels}$
$n_{\infty }\left(V\right)={\left(1+\exp(\left(u_{n}-V\right)/s_{n})\right)}^{-1}$	Steady-state potassium ion activity
$J_{mem}=-\left(\alpha I_{Ca}/V_{cyt}+k_{PMCa}Ca\right)$	Calcium influx into the cell
$J_{er}=k_{SerCa}Ca-p_{leak}\left(Ca_{er}-Ca\right)$	Calcium influx into the ER
$J_{m}=k_{uni}Ca-k_{NaCa}\left(Ca_{m}-Ca\right)$	Calcium influx into the mitochondria
$J_{PFK}=v_{PFK}\frac{w_{1110}+k_{PFK}\sum_{i,j,l\in \left\{0,1\right\}}w_{ij1l}}{\sum_{i,j,k,l\in \left\{0,1\right\}}w_{ijkl}}$	PFK activity according to [33]
$w_{ijkl}=\frac{{\left(AMP/K_{1}\right)}^{i}{\left(FBP/K_{2}\right)}^{j}{\left(F6P/K_{3}\right)}^{k}{\left(ATP/K_{4}\right)}^{l}}{f_{13}^{ik}f_{23}^{jk}f_{41}^{il}f_{42}^{jl}f_{43}^{kl}}$	Weight factor according to [33]
$AMP=ADP^{2}/ATP$	Amount of adenosine monophosphate
$J_{PDH}=v_{PDH}s_{\infty }\left(Ca_{m}\right)\sqrt{FBP}$	Pyravate dehydrogenase reaction rate
$s_{\infty }\left(Ca_{m}\right)=Ca_{m}/\left(K_{PDH}+Ca_{m}\right)\;$	Feedback on pyravate dehydrogenase by calcium in the mitochondria
$ATP=\frac{1}{2}\left(A_{tot}+\sqrt{-4ADP^{2}+{\left(A_{tot}-ADP\right)}^{2}}-ADP\right)$	Amount of ATP

**Table A3:** Parameter set for the reference model, taken from [36] and the reference therein.

$C_{m}=5300\;\mathrm{fF}$	$k_{tt}=1\;\text{µ}M$	$k_{PFK}=0.06$
$g_{Ca}=1000\;\mathrm{pS}$	$k_{td}=26\;\text{µ}M$	$K_{1}=30\;\text{µ}M$
$g_{K}=2700\;\mathrm{pS}$	$f_{Ca}=0.01$	$K_{2}=1\;\text{µ}M$
$g_{K\left(Ca\right)}=700\;\mathrm{pS}\;\left(2.2.1\right),\;50\;\mathrm{pS}$ (else)	$\alpha =5.18\times 10^{-18}\;µ\mathrm{mol}/\left(\mathrm{fA}\cdot \mathrm{ms}\right)$	$K_{3}=5\times 10^{4}\;\text{µ}M$
$g_{K\left(ATP\right)}=25000\;\mathrm{pS}$	$V_{cyt}=1.15\times 10^{-12}\;l$	$K_{4}=1\times 10^{3\;}µM$
$V_{Ca}=25\;\mathrm{mV}$	$k_{PMCA}=0.2\;{\mathrm{ms}}^{-1}$	$f_{13}=0.02$
$V_{K}=-75\;\mathrm{mV}$	$k_{SERCA}=0.4\;{\mathrm{ms}}^{-1}$	$f_{23}=0.2$
$V_{K}=-20\;\mathrm{mV}$	$p_{leak}=2\times 10^{-4\;}{\mathrm{ms}}^{-1}$	$f_{41}=20$
$s_{m}=12\;\mathrm{mV}$	$k_{uni}=0.4{\;\mathrm{ms}}^{-1}$	$f_{42}=20$
$u_{n}=-16\;\mathrm{mV}$	$k_{NaCa}=0.001\;{\mathrm{ms}}^{-1}$	$f_{43}=20$
$s_{n}=5\;\mathrm{mV}$	$\sigma_{m}=100$	$v_{PDH}=0.009\;\text{µ}M/\mathrm{ms}$
$\tau_{n}=20\;\mathrm{ms}$	$\sigma_{er}=31$	$K_{PDH}=200\;\text{µ}M$
$k_{d}=0.5\;\text{µ}M$	$J_{GK}=0.0015\;\text{µ}M/\mathrm{ms}\left(2.2.1\right),\;J_{GK}=0.001\;\text{µ}M/\mathrm{ms}\;\left(\mathrm{else}\right)$	$\tau_{a}=3\times 10^{5}\;\mathrm{ms}$
$k_{dd}=17\;\text{µ}M$	$v_{PFK}=0.01\;\text{µ}M/\mathrm{ms}$	$A_{tot}=3\times 10^{3}\;\text{µ}M$
